# Determining the Effect of Non-Thermal Plasma on the Transmembrane Kinetics of Melittin through Molecular Explorations

**DOI:** 10.3390/biom14101207

**Published:** 2024-09-25

**Authors:** Yanxiu Cui, Tong Zhao, Yanxiong Niu, Xiaolong Wang, Yuantao Zhang

**Affiliations:** School of Electrical Engineering, Shandong University, Ji’nan 250061, China; cuiyanxiu@mail.sdu.edu.cn (Y.C.); niuyx@mail.sdu.edu.cn (Y.N.); wangxiaolong@sdu.edu.cn (X.W.); ytzhang@sdu.edu.cn (Y.Z.)

**Keywords:** melittin, cold plasma, membrane lipid bilayer, synergistic effect, molecular dynamics

## Abstract

Non-thermal plasma (NTP) synergistic anticancer strategies are a current hotspot of interest at the intersection of plasma biomedicine. Melittin (MEL) has been shown to inhibit cancer in many malignant tumors; however, its clinical application is controversial. Therefore, the transmembrane process and mechanism of MEL activity in different cell systems were studied and the combination of MEL and NTP was proposed in this paper. The results showed that the electrostatic attraction between MEL and the lipid bilayer contributes to the stable orientation of MEL on the membrane surface. In addition, sialic acid overexpression affects the degree to which MEL binds the membrane system and the stability of the membrane structure. The use of NTP to reduce the dosage of MEL and its related nonspecific cytolysis activity has certain clinical application value. The results of this study provide theoretical support for improving the clinical applicability of MEL and contribute to the further development of plasma biomedicine.

## 1. Introduction

Non-thermal plasma (NTP) is an ionized gas composed of ions, electrons, free radicals and reactive neutral substances [1] Due to its advantages, including high reactivity, low energy consumption and environmental friendliness, NTP has been widely used in many fields, such as biomedicine and environmental protection [2,3,4,5,6]. Cancer is a major challenge and threat to human life and health. The effects of NTP on cancer cells and the underlying mechanism have been continuously explored. Researchers generally believe that RONS (reactive oxygen and nitrogen species) [7,8], which are active substances produced by NTP treatment, change the biological properties of cells or tissues [9]. Compared to healthy cells, cancer cells exhibit greater sensitivity [10] and are more susceptible to NTP treatment and oxidative damage; however, the extent of damage varies with the dose of NTP, and cancer cell therapy often requires a greater intensity of action and a longer duration than that of other treatments [11]. Combining other drugs or anticancer strategies to achieve synergistic therapy is a developing trend in plasma cancer biomedicine.

As a naturally occurring cytolytic peptide, melittin (MEL) directly kills tumor cells in an effective manner and exhibits a variety of immunomodulatory functions; thus, the potential use of MEL in cancer therapy has attracted considerable attention [12] due to the low likelihood of drug resistance [13]. Zhang et al. observed that subcutaneous injection of different doses of melittin into nude mice with non-small-cell lung cancer inhibited the invasion and migration of lung cancer cells to varying degrees [14]. Zhou et al. demonstrated the role of melittin in inducing the apoptosis of human esophageal cancer cells [15]. The unique amphiphilic structure of melittin lays a foundation for its ability to destroy the cell membrane system [16]. Mihajlovia et al. reported that melittin directly interacts with the membrane [17], interfering with the integrity of the phospholipid bilayer by forming transmembrane pores or ion channels; this process is accompanied by the leakage of ions and molecules and an increase in membrane permeability. In addition, when melittin reaches the intracellular environment, it can act on the inner organelle membrane in a similar manner to induce biochemical cell apoptosis [13]. MEL effectively induces apoptosis and mitochondrial destruction and inhibits cancer cell invasion [18]. Growing evidence indicates that melittin exhibits powerful antitumor effects; however, the clinical application of MEL is a major challenge due to its rapid degradation in the blood and nonspecific toxicity at high doses. Therefore, further research is needed to improve the therapeutic effectiveness of MEL.

During NTP treatment, the main cell component that undergoes chemical modification by plasma reactive species is the cell membrane, and the anticancer mechanism of MEL is also associated with the destruction of cell membrane integrity. In this case, combining plasma technology with MEL IV seems advantageous. The oxidative modification of the membrane structure by NTP induced changes in membrane permeability, laying a foundation for the process that disrupts the MEL membrane. The dual action of MEL and plasma reactive species in and out of the membrane reduced the therapeutic dose of MEL and its related nonspecific toxic effects while reducing the therapeutic intensity and duration of NTP. Although Shaw P’s team [19] has conducted research to support this possibility, the interaction mechanism involved has not been fully investigated, and the mechanism by which melittin destroys the membrane surface is unclear. Considering the positive nature of melittin, we targeted the negatively charged sialic acid molecules on the surface of the membrane to study its interaction mechanism; this was performed to clarify how the surface components of cancer cell membranes dynamically affect MEL. Sialic acid is usually located at the ends of glycoproteins and glycolipids on the cell surface [20] and is an important component of membrane surface receptors. Sialic acid is abnormally highly expressed in various inflammatory diseases and in the context of tumor transformation and deterioration [21] and is widely recognized as a biological marker of inflammation and cancer [22,23,24]. Examining the influence of negatively charged sialic acid on the disturbance of the MEL membrane is helpful for determining the mechanism by which MEL destroys membranes in a real membrane environment.

In the complex environment of real cell membranes, it is very difficult to fully achieve the goal by relying on experimental measurements alone. Molecular dynamics (MD) simulations involve the use of computers to study the dynamic transformation process of systems composed of atoms and molecules; these simulations are widely used to study the biological process of cell membrane interfaces, which can provide powerful support to experimental processes. Ghasemitarei M [9] et al. highlighted the application and potential of classical molecular dynamics simulations in studying the effects of NTP on biofilm systems and their interactions. For example, in a study by Yusupov’s team, they used MD simulations to investigate the oxidative damage of NTP-generated RONS on hyaluronic acid (HA) and cluster of differentiation 44 (CD44) and their effect on their binding affinity, and the dissociation-free energy spectrum obtained through simulations showed that the oxidation of CD44 and HA weakened their interaction. This inhibited the signaling pathway for cancer cell proliferation, which strongly complemented their experimental inference that reducing the interaction between HA and CD44 through NTP treatment may disrupt the signaling pathway that drives tumor progression [25]. In this study, we used molecular dynamics (MD) simulations to describe the interaction mechanism of melittin and the influence of membrane surface components to provide a reference for the effectiveness of collaborative plasma therapy. In this study, the internal mechanism of the MEL membrane disturbance process was studied innovatively using negatively charged sialic acid on the membrane surface as the starting point. Then, the change in the transmembrane potential energy of MEL before and after NTP activity was compared to explore the feasibility of combined NTP and MEL cancer therapy. Overall, our computational results further reveal the dynamic behavior of MEL influenced by membrane surface components and suggest a new synergistic therapy for future cancer treatment, which may improve the clinical applicability of melittin.

## 2. Methods and Materials

### 2.1. Simulation Setup and Model Introduction

**Figure 1:**
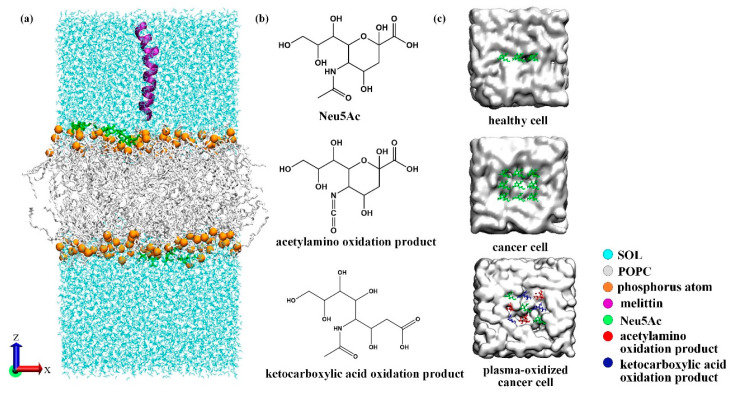
(**a**) Schematic diagram of the membrane system; (**b**) structural formula of sialic acid molecules and their plasma oxidation products; (**c**) schematic diagram of the distribution of sialic acid molecules and their oxidation products on the membrane surface (xy plane) at the initial stage of modeling.

In this study, 2-oleoyl-1-palmitoyl-sn-glycero-3-phosphocholine (POPC) was selected as the structural skeleton of cell membranes [26]. Based on the difference in sialic acid content on the surface of healthy cells and cancer cells, a model system was established to study the effect of plasma oxidation on the transmembrane transfer of melittin; this experiment involved replacing sialic acid molecules on the surface of cancer cells with sialic acid oxidation products in equal quantities. PackMol software (Version 18.169) [27] was used to generate the initial configuration of the stable membrane system. A total of 128 phospholipids were composed of bilayer structures, and the membrane system was prebalanced by 200 ns. In this paper, the chain A coordinate of the crystal structure of melittin (PDBID:2MLT) was used to set the system, hereinafter referred to as MEL. Using the phospholipid bilayer 200 ns equilibrium model as the initial structure, MEL was placed in the water phase approximately 1.0 nm above the head group region of the phospholipid bilayer, as shown in Figure 1a. Simple Point Charge water (SPC) molecules [28] were used to represent the aqueous environment around the membrane and MEL. Appropriate amounts of Na^+^ and Cl^–^ ions were added to maintain the electroneutrality of the membrane system. After the energy of the model system was minimized, 50 ns NPT equilibrium was performed with a time step of 2 fs. A Nose–Hoover temperature controller [29] was used to control the temperature at 310 K. The semi-isotropic pressure coupling of the Parrinello–Rahman barometer [30] was used to maintain the pressure at 1 bar. The position of the MEL was restricted to avoid shifting behavior in the equilibrium stage of the system.

Figure 1 shows a legend that applies to all graphics in the entire paper.

This is the first study on the reaction characteristics of plasma reactive species (with n–acetylneuraminic acid as the main sialic acid structure) on the surface of human cells, and their main oxidation products, including acetylamino oxidation products and ketocarboxylic acid oxidation products, were obtained. To simplify the simulation system, we arranged 3 Neu5Ac (n–acetylneuraminic acid) molecules on the surface of healthy cells and 9 Neu5Ac molecules on the surface of cancer cells in the initial modeling stage and represented the cancer cell system after plasma oxidation in the form of Neu5Ac molecules, acetylamino oxidation products, and ketocarboxylic acid oxidation products in a 3:3:3 ratio. The specific arrangement is shown in Figure 1c.

### 2.2. MD Trajectory Analysis

The utility program that accompanied the GROMACS software (2020.6) package [31] was used to analyze the MD trajectory. All parameters were analyzed and calculated in the equilibrium part (past 10 ns) of the corresponding MD trajectory, including electrostatic and van der Waals effects, hydrogen bonds, structural parameters of the membrane system, density distribution, etc. The image of the membrane binding process was rendered by VMD [32], and the data drawing was completed by Origin [33].

The formula for calculating the membrane surface tension is as follows:

$$\gamma \left(t\right)=\frac{L_{Z}}{n}\langle P_{zz}\left(t\right)-\frac{P_{\mathrm{xx}}(t)+P_{yy}(t)}{2}\rangle$$

where n represents the number of interfaces of the system, $L_{Z}$ is the length of the Z direction of the simulation box, $P_{\mathrm{xx}}$, $P_{yy}$ and $P_{zz}$ are the pressures in the x, y and z directions, respectively. 〈 〉 represents the time average.

### 2.3. Umbrella Sampling

To determine the promoting effect of surface components of the plasma oxide film on melittin transmembrane transport, we applied umbrella sampling (US) to calculate the transmembrane-free energy. The initial model is the MEL-phospholipid bilayer model after adding reactive species and completing the equilibrium simulation. In the 9 nm space along the membrane normal direction (Z-axis direction), 300 windows were defined, and the umbrella-shaped sampling windows were spaced 0.03 nm equidistant along the reaction coordinates, which was enough to cover the entire membrane architecture. For each simulation system, a tractive force of 1000 kJ·mol^−1^·nm^−2^ was applied in the membrane normal direction (Z-axis direction) at a traction rate of 0.003 nm/ps, so as to more clearly analyze the dynamic process of MEL across the membrane under each window. The force constant is set in accordance with Irudayam S J [34]. The weighted histogram analysis method (WHAM) was used in GROMACS to calculate the mean free energy of melittin across membranes.

## 3. Results 

### 3.1. Analysis of Physicochemical Properties of Melittin

**Figure 2:**
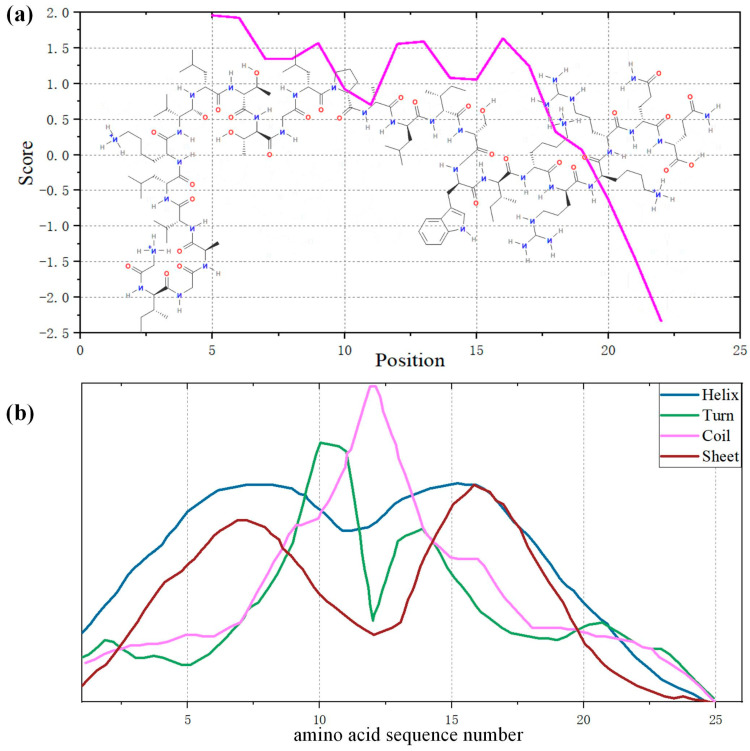
(**a**) Analysis diagram of the molecular structure and hydrophilic and hydrophobic properties of MEL (a score less than 0 indicates hydrophilicity); (**b**) the presence of secondary structures at different positions of the MEL amino acid sequence.

MEL is a polypeptide composed of 26 amino acid residues. The amino acid symbol mapping table and its properties are shown in Table 1. According to the analysis of the ExPASy series database [35], the relative molecular weight of MEL is 2847, and its theoretical isoelectric point is 12.02. MEL is an unstable alkaline polypeptide with an instability coefficient of 44.73. The molecular structure and hydrophilicity of the MEL crystals are shown in Figure 2a, with a total average hydrophilic index of 0.273 and a lipid solubility coefficient of 135. MEL is a lipophilic protein with membrane activity that can directly dissolve the phospholipid membrane of cells. Figure 2a shows that the first 20 amino acid residues from the n–terminus of melittin are mainly hydrophobic, the 6 amino acid residues from the c–terminus are mainly hydrophilic, and their amphiphilicity indicates that MEL can be both dissolved in water and naturally bound to the membrane. This is precisely the underlying premise of MEL’s membrane-binding mechanism, which primarily involves its ability to interact with the lipid bilayer, leading to membrane destabilization rather than relying on passive diffusion through the membrane.

The secondary structure prediction analysis of the polypeptide is shown in Table 2, indicating that the α-helix is the main component of the secondary structure of the polypeptide. Figure 2b shows the presence of secondary structures at different positions of the MEL amino acid sequence. The helix–crimp–helix structure of MEL was observed, and the crimp site was located at amino acid residues 10 to 15, which laid the foundation for the “U–shaped” conformation in the transmembrane process.

### 3.2. Membrane Lipid–Melittin Interaction

**Figure 3:**
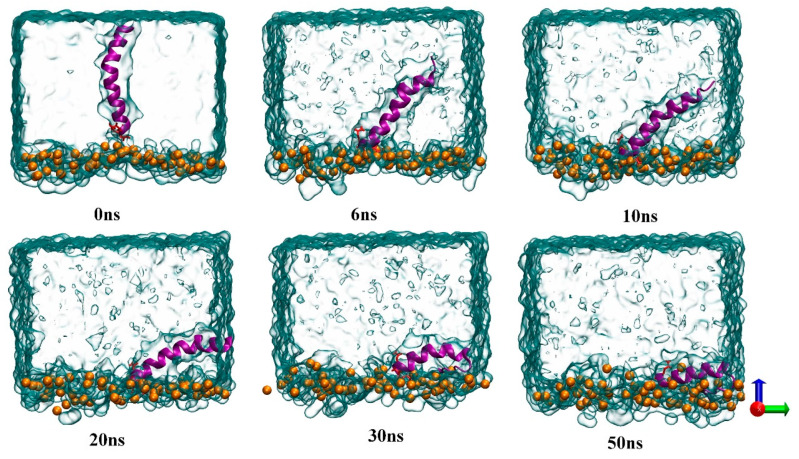
Image of the kinetic process of melittin binding to the membrane system under conventional MD simulation.

**Figure 4:**
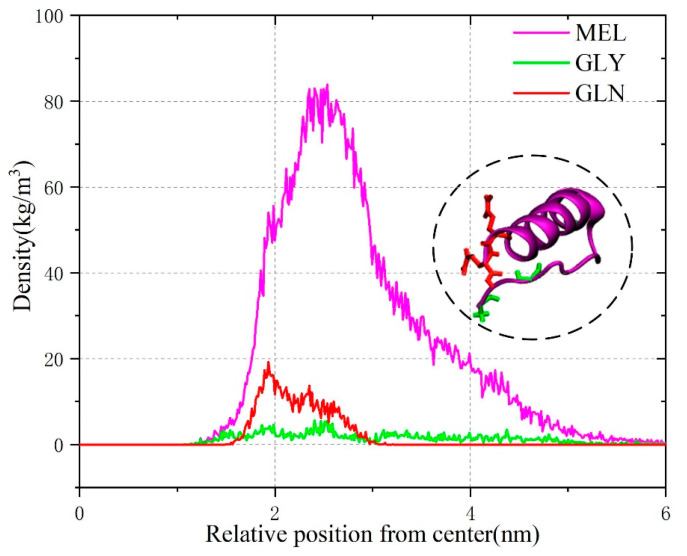
Density distribution of MEL and its residues GLY and GLN. The illustration shows the MEL conformation at 50 ns.

In this paper, we first performed molecular simulations to visualize the binding process of melittin to electrically neutral liposomes. In this conventional MD simulation, the hydrophilic c–terminus of melittin is placed close to the phospholipid bilayer head region, and the hydrophobic n–terminus faces water. This initial setup is intended to examine the amphiphilic structure of MEL in order to better understand the role of this structure in the mechanism of MEL-induced membrane disruption. Figure 3 shows the structural changes in melittin that occurred during the binding process from the aqueous phase to the lipid bilevel surface under conventional MD simulation. The GLN residue of melittin is represented by a red rod to distinguish the hydrophobic and hydrophilic ends of MEL, and GLN is close to the c–terminus. After the simulation began, MEL quickly contacted the phospholipid head region, and since the c–terminus residue was mainly hydrophilic and positively charged, it adhered to the phospholipid head region. The hydrophobic N-terminus showed an irregular bending phenomenon as the simulation time progressed, and it gradually approached the phospholipid bilayer and was inserted into the hydrophobic region below the head region defined by lipid phosphorus atoms. Multiple simulation results showed that MEL maintained a flat orientation on the surface after binding with electroneutral liposomes. Due to the new orientation of the peptide around its spiral axis, the hydrophobic n–terminus was oriented toward the lipid tail, MEL exhibited a “U–shaped” conformation, and the peptide conformation was stable in the late simulation period. This conclusion is further explained by a density distribution diagram formed for the polypeptide and its residues, which is shown in Figure 4. GLY residues at the hydrophobic n–terminus undergo continuous dynamic changes from the initial conformation toward phospholipids, while GLN residues fluctuate significantly because the residues adhere to the hydrophilic region of phospholipids.

**Figure 5:**
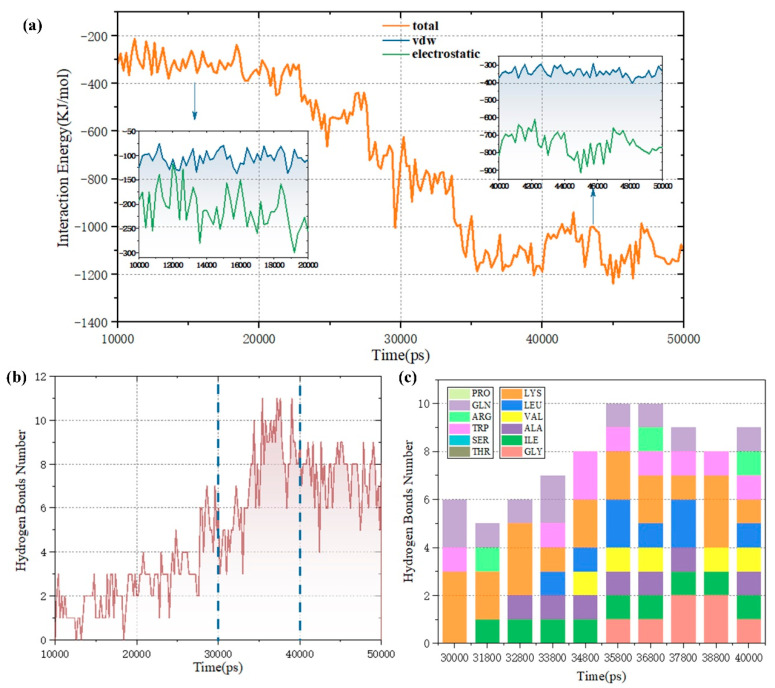
(**a**) The interaction energy of the MEL and POPC lipid bilayer. The illustration shows that the simulated interactions at 10–20 ns and 40–50 ns can be divided into vdw terms and electrostatic terms. (**b**) Hydrogen bond formation between MEL and the POPC lipid bilayer. (**c**) The formation of hydrogen bonds between MEL residues and lipids during the period of 30–40 ns.

The interaction energy between MEL and the phospholipid bilayer was calculated to elucidate the dynamic change in peptide conformation on the phospholipid bilayer surface, which was divided into electrostatic and van der Waals (vdW) interactions before and after simulation. As shown in Figure 5a, according to the distribution curve, the peptide conformation begins to stabilize at approximately 35 ns, which is consistent with the results shown in Figure 3. After 30 ns, the peptide conformation on the surface of the membrane system basically adopts a “lying orientation”, and the interaction can remain relatively stable. During the interaction, the fluctuation degree of the interaction energy curve is mainly affected by the electrostatic action. As shown in the inset of 40–50 ns in Figure 5a, after the peptide conformation becomes relatively stable, the average electrostatic energy contribution during this period is 715.571 kJ/moL, while the van der Waals energy contribution averages 335.077 kJ/kJ/moL. The electrostatic contribution is approximately twice that of the van der Waals interaction. Therefore, electrostatic action may play a dominant role in the binding process of MEL to the membrane system, and the initial binding to the double-layer surface may be driven by the electrostatic attraction between the MEL residues and phospholipid molecules, as shown in Figure 5b,c. Notably, the positively charged residue LYS participated in hydrogen bond formation for more than half of the simulated trajectories, showing a high hydrogen bond tendency. However, one LYS is located at the hydrophobic n–terminus of MEL and contacts many lipid tails, which may lead to membrane surface disturbance and induce membrane pore formation; this result is consistent with the conclusion of Manna M et al. [36], and the interaction contributes to the stable orientation of melittin on the membrane surface.

### 3.3. Effect of Sialic Acid on the Transmembrane Behavior of MEL

**Figure 6:**
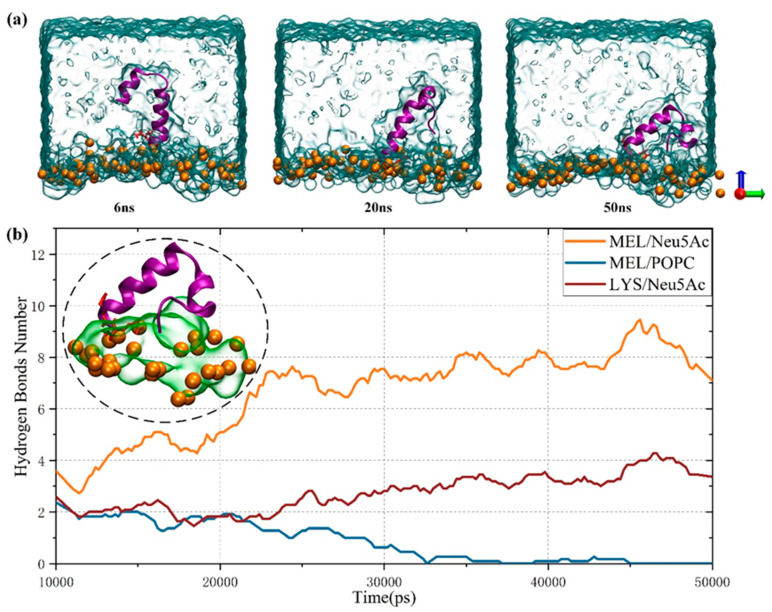
(**a**) Schematic diagram of the dynamic process by which MEL binds to the membrane surface of cancer cells; (**b**) hydrogen bond formation among components on the surface of the cancer cell membrane. The illustration shows the adsorption of MEL (purple) with a Neu5Ac molecule (green) and a lipid phosphorus atom (yellow) on the surface of the cancer cell membrane at 50 ns.

Importantly, membrane perturbation induced by MEL transmembrane results from the adaptation and stabilization of peptides in membrane systems. An important issue is that some changes in peptide conformation may be accompanied by interactions with membrane surface components. Therefore, the transmembrane similarities and differences in MEL in cancer cells and healthy cells were compared in this paper to study the influence of sialic acid content on the transmembrane behavior of MEL. Figure 6a shows the binding of MEL to the membrane surface of cancer cells. The membrane adsorption behavior of MEL was more greatly affected by the increase in sialic acid content on the membrane surface of cancer cells than healthy cells (Figure 3). Shortly after the simulation began, MEL tended to adopt a “U–shaped” conformation. The degree of MEL binding on the surface of the cancer cell membrane was low, the binding state of the cancer cell membrane surface showed a slight “hanging” phenomenon until 50 ns, and some residues remained in the water phase, which may result from the proximity attraction of the Neu5Ac molecule. As shown in Figure 6b, the hydrogen bond attraction between the positively charged MEL residue and the sialic acid molecule is strong, which may explain the large fluctuation of MEL observed in the simulation process. The hydrophilic end of MEL closely contacts the lipid phosphorus atom, and the N-terminus is affected by the special residue LYS, which contacts Neu5Ac molecules.

Furthermore, we calculated the average time for several parameters of the membrane system, as shown in Table 3, to evaluate the potential of melittin to induce membrane pore formation in different membrane systems. The membrane surface tension gradually increases with the peptide binding process, which is accompanied by the thinning and expansion of the phospholipid membrane; this “expansion” in turn leads to an increased likelihood of membrane defects. MEL interacts with the phospholipid layer to alter the physical properties of the membrane, thereby impacting its stability and functionality. Interestingly, melittin is not specific across membranes. Although the binding of MEL to membranes in cancer cell systems with increased sialic acid content may simultaneously lead to a “hanging” phenomenon, the increased sialic acid content also impacts the stability of the membrane system, which can be supported by the data in Table 3. It shows that the relative change in surface tension of the cancer cell membrane system before and after MEL membrane binding increased by 3% compared to healthy cells, while the relative change in membrane area expanded by as much as 69.74 times. In this case, considering the combination of the plasma effects with reduced MEL levels to mitigate potential toxicity associated with high concentrations holds certain clinical application potential, which is the main focus of our next section.

### 3.4. Synergistic Effect of Plasma and Melittin across Membranes

**Figure 7:**
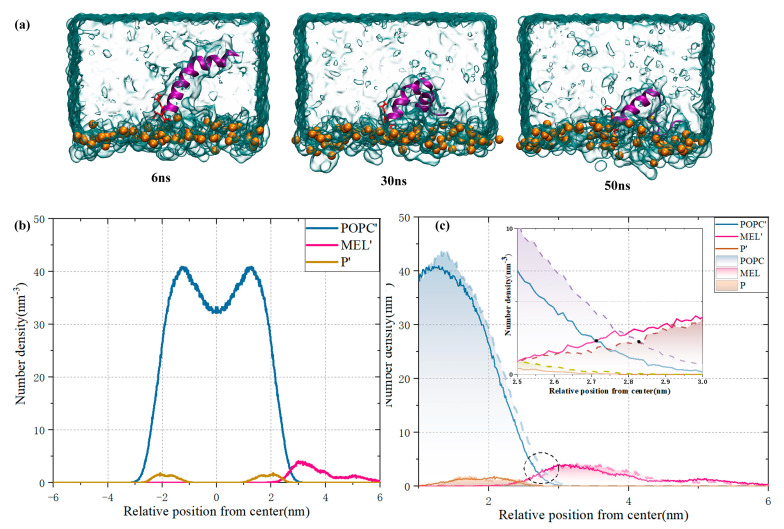
(**a**) Image of the membrane binding dynamics of MEL and cancer cells in the presence of transmembrane potential; (**b**) the number density distribution along the bilayer normal in the presence of the transmembrane potential; (**c**) local map of the number density distribution, in which the band represents a membrane system with a transmembrane potential; otherwise, the membrane system does not contain a transmembrane potential.

In the actual biological environment, the unbalanced distribution of positive and negative ions inside and outside the cell leads to a potential gradient inside and outside the cell. As plasma acts on biological tissues, changes in the spatial distribution of reactive species in the membrane system may enhance the transmembrane potential due to changes in membrane permeability or the presence of ion channels. The transmembrane potential affects the formation of cell membrane pores and carrier entry [37]. Therefore, we added 30 Na^+^ ions to the extracellular environment to simulate the effect of the membrane potential on MEL membrane binding and explore the feasibility of the synergistic effect of plasma and MEL. It is worth noting that this is primarily a qualitative analysis. As shown in Figure 7, in the presence of the transmembrane potential, the hydrophobic n–terminus of MEL gradually approached the membrane surface due to electrostatic attraction as the simulation progressed, and its binding degree improved compared with that in Figure 6. Additionally, the irregular bending degree of the n–terminus increased. Based on the comparison of Figure 7b,c, the number density distribution along the double layer normal with or without the presence of transmembrane potential indicated that the MEL density distribution is closer to the lipid double layer with the presence of transmembrane potential, and the membrane binding interaction decreases the thickness of the local membrane. In the limited time simulation, the formation of membrane pores and the entire penetration of MEL were not observed; however, in the actual plasma treatment process, the infiltration of reactive species led to a continuous increase in the electric potential difference between inside and outside the cell. This process provided an opportunity for peptides to penetrate into the cell, and a low dose of MEL led to cytotoxic effects. The combination of MEL and NTP to reduce the dosage of MEL has a certain clinical value. The literature [19] has demonstrated the potential of NTP synergies in ovum experiments and in vitro.

**Figure 8:**
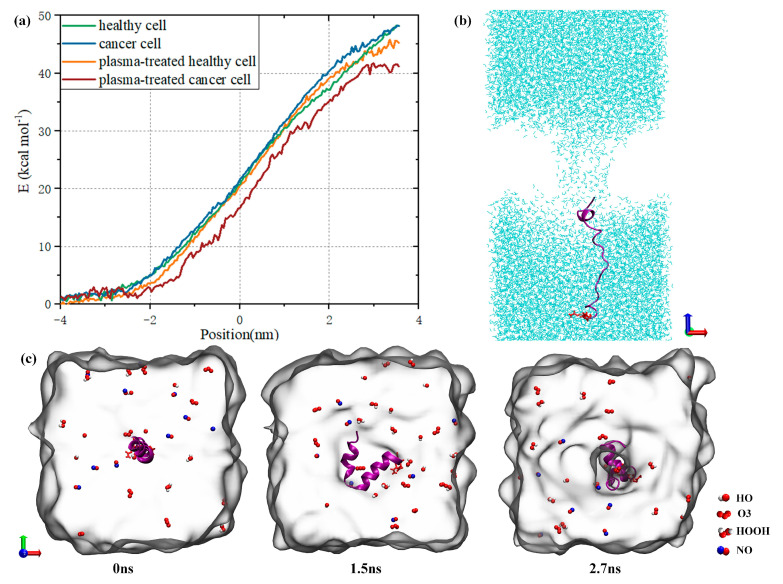
(**a**) Changes in the umbrella potential energy in different membrane systems; (**b**) schematic diagram showing the transmembrane completion of melittin in cancer cells; (**c**) image of the pore formation and transmembrane processes of reactive species in the oxidized cancer cell system.

Furthermore, we performed an umbrella sampling simulation to elucidate the synergistic effect of plasma treatment on the entry of MEL into cells. As shown in Figure 8a, the transmembrane behavior of MEL changes in both healthy and cancer cell systems due to the different contents of sialic acid on the membrane surface and its influence on membrane stability; however, the final transmembrane potential energy of MEL is basically the same. In contrast, the transmembrane potential energy of MEL decreases in the membrane system after plasma treatment. The transmembrane barrier of oxidized cancer cells decreased by approximately 7 kcal moL^−1^, which was 2.8 times that of healthy cells under the same conditions. This result demonstrates the effectiveness of plasma-assisted treatment, in which MEL is more likely to penetrate into cancer cells than healthy cells; in addition, MEL may help induce the apoptosis of cancer cells without damaging healthy cells since lower doses of MEL exert cytotoxic effects. MEL will form transmembrane pores under umbrella sampling pull, which enter the cell membrane in the form of a chain, as shown in Figure 8b,c. This transmembrane process is accompanied by leakage of water molecules, etc. As MEL transitions from one side of the cell to the other side, its hydrophilic c–terminus is attracted by the extracellular water environment and reaches the other side of the phospholipid first. We added 10 reactive species of HO, HOOH, O_3_ and NO (the reactive species selected here are some of the more common reactive ingredients of plasma products [38]) to the extracellular environment and observed that these species can enter the cell through the membrane pore, which provided another channel for the plasma reactive species to enter the cell. The reactive species could accumulate through entering the cell, thus causing further oxidative damage to the cell. It is worth noting that the research here focuses on whether RONS can pass through the transmembrane pores formed during the MEL membrane bonding process, and the stability of RONS during the transmembrane process has not been studied. Due to the difference in the composition of cancer cells and healthy cells and the greater sensitivity of cancer cells to oxidative stress [9], healthy cells should be less susceptible to synergistically induced oxidative nitrification and membrane pore formation under the same conditions during the synergistic treatment of cancer with NTP and MEL; relatively speaking, this process can induce apoptosis of cancer cells while reducing the toxic effect on healthy cells.

## 4. Discussion

The combined therapy of NTP and MEL presents a promising approach to overcoming the nonspecific toxicity typically associated with MEL. The key advantage lies in the differential effect this combination has on cancer cells versus healthy cells. The plasma treatment generates reactive oxygen and nitrogen species (RONS), which increase the permeability of cancer cell membranes, allowing MEL to act more efficiently in inducing cell death. As a result, the treatment selectively targets cancer cells, offering a therapeutic window to minimize damage to healthy tissue. This dual action—enhanced membrane disruption by MEL and increased susceptibility of cancer cells due to plasma-induced oxidative stress—highlights the potential for more targeted, less toxic cancer therapies.

Despite laying the foundation for innovative cancer treatments utilizing the synergistic effect of plasma and MEL, it is undeniable that further research is needed. Can sialic acid on the membrane surface serve as a target for localized cancer treatment using plasma? Could specific delivery systems be developed to concentrate RONS at the tumor site? How can damage to healthy cells be further minimized? In cases where cancer cells are located beneath the skin, how can minimally invasive techniques or other methods be used to deliver plasma precisely for maximum therapeutic effect? We acknowledge that these approaches are still far from widespread clinical application, but they represent key directions for future research.

## 5. Conclusions

This study explored the mechanism underlying MEL activity on healthy and cancer cells with different membrane surface sialic acid contents from a molecular perspective. Due to the nonspecific toxicity of MEL, the potential application of non-thermal plasma and MEL in synergistic cancer treatment was further investigated. The results indicate that the unique amphiphilic structure of MEL increases its ability to adapt to the water lipid bilayer, and MEL tends to adopt a “lying flat” orientation on the membrane surface. MEL’s membrane-disrupting mechanism primarily involves its ability to interact with the lipid bilayer. The electrostatic attraction between the MEL cationic residue LYS and the phospholipid bilayer is a cause of membrane disturbance and induces membrane pore formation. With increasing sialic acid content on the surface of cancer cell membranes, the attraction of sialic acid molecules to MEL increases. Along with the thinning and expansion of phospholipid membranes, the degree of MEL binding to the membrane surface is slightly reduced. The stability of the membrane system and the degree of MEL membrane binding both changed; therefore, the synergistic effect of NTP and MEL has application potential.

In the process of plasma treatment, the continuous infiltration of reactive species leads to an increase in the transmembrane potential, which provides an opportunity for MEL to exhibit transmembrane behavior. As shown by the umbrella sampling results, compared to healthy cells, cancer cells are more sensitive due to oxidative stress; in addition, the decrease in transmembrane potential energy of cancer cells after plasma treatment is approximately 2.8 times that of healthy cells. The formation of MEL transmembrane pores can promote the infiltration of reactive species. Our results show that NTP combined with MEL did not produce a simple additive effect on cancer treatment, as a complex synergistic effect was observed; this effect reduced the permeability and integrity of phospholipid cells, promoted the entry of reactive species and MEL into cells, and damaged cellular components to induce apoptosis. Due to the differential expression between cancer cells and healthy cells, the ability to successfully induce apoptosis of cancer cells while ensuring the survival of healthy cells is increased when the degree of damage to healthy cells is low under the same treatment conditions. In conclusion, the synergistic effect of NTP and MEL has great application prospects in MEL-based cancer therapy and helps reduce the nonspecific toxic side effects of MEL.

## Figures and Tables

**Table 1:** Amino acid symbol comparison table and properties. “O” means hydrophobic, “I” means hydrophilic, “M means neutral” and “l+” means it has a positive charge.

Name	Symbol	Properties
Glycine (Gly)	G	I/M
Isoleucine (Ile)	I	O/M
Alanine (Ala)	A	O/M
Valine (Val)	V	O/M
Leucine (Leu)	L	O/M
Lysine (Lys)	K	I/l+
Proline (Pro)	P	O/M
Threonine (Thr)	T	I/M
Serine (Ser)	S	I/M
Tryptophan (Trp)	W	O/M
Arginine (Arg)	R	I/l+
Glutamine (Gln)	Q	I/M

**Table 2:** The proportion of MEL secondary structure prediction analysis.

α-Helix	β-Turn	Random Coil	Extension Chain
53.85%	26.92%	11.54%	7.69%

**Table 3:** Time average values of several parameters of the membrane system.

Parameter	Healthy Cell	Cancer Cell
Surface tension/nN × m^−1^	5.453784 (1.679062)	5.464252 (1.59166)
Membrane area/nm^2^	39.67764 (39.66392)	40.83215 (39.86161)
Membrane thickness/nm	3.91129 (3.99502)	3.93175 (4.00525)

Note: The values in brackets are the equilibrium parameters corresponding to MEL before membrane binding.

## Data Availability

Data will be made available on request.
